# Using Structural Equation Modeling to Examine Pathways Between Environmental Characteristics and Perceived Restorativeness on Public Rooftop Gardens in China

**DOI:** 10.3389/fpubh.2022.801453

**Published:** 2022-02-24

**Authors:** Zhanglei Chen, Kar Kheng Gan, Tiejun Zhou, Qingfeng Du, Mingying Zeng

**Affiliations:** ^1^School of Architecture and Urban Planning, Chongqing University, Chongqing, China; ^2^TCL Studio, Adelaide, SA, Australia; ^3^Chinese Society for Urban Studies, Beijing, China; ^4^School of Civil Engineering and Architecture, Southwest University of Science and Technology, Mianyang, China

**Keywords:** public rooftop garden, restorative environment, perceived sensory dimensions, mediating mechanism, structural equation modeling

## Abstract

As a kind of informal green space more closely related to the built environment, public rooftop gardens (PRGs) are novel green open space and important salutogenic resource for urban residents. It is one of the most easily accessible method for urban residents to be in contact with outdoor or natural elements from the context of high-altitude living. Given its potential health benefits to city dwellers, existing empirical studies are heavily focused on immediate recovery through visually accessing PRGs (through windows), neglecting the possibility of using PRGs physically as a place of interaction. This paper hypothesizes usage patterns will mediate the associations between the environmental characteristics of PRG and users' restoration. This is done through inputting data from 12 typical samples of PRG in Chengdu, China into structural equation model (SEM). Combining the concept of Perceived Sensory Dimensions (PSD) and Perceived Restorativeness Scales (PRS) with the usage patterns of the above samples, this study aims to examine the correlation of environmental characteristics, usage pattern and restoration, in which identify their relative importance in the context of PRGs. Through serials of numerical tests on the model, the study shows that out of the 20 theoretical pathways constructed by the environmental characteristics (x)–usage patterns (m)–restorative effect (y), only 14 forms a significant correlation. In addition, out of all PSDs, social, serene, refuge, space and nature dimensions are induced into restorative effects through four patterns of use: retreat, nature touch, interpersonal interaction and family-bonding activities. The findings also show that social and family-bonding are the most influential independent and mediating variables respectively in achieving restorative effects in the PRG. This study reveals important findings about how usage patterns mediate the association between the PSD and PRS of users. And it also has generated practical implications on how we can design public rooftop gardens from the perspective of restoration, which could potentially be the key to the future survival and development of PRGs in urban environments.

## Introduction

Over the past two decades, the gradual increase in urban density has becoming a common denominator in the world's urban development. Although cities account for only about 3% of land area globally, they are home to more than half of the world's population. According to the World Health Organization (WHO) 2014 prediction, more than 70% of the world's population will live in cities in the next 30 years. Hence, urban areas that are heavily populated will be facing serious environmental degradation, which reduces all the ecosystem services that is part of the natural environment. Consequently, the diminished of these large number of biophysical and cultural services that contribute to health and well-being are going to have considerable impact on the mental and psychological health of the general populations (1–4). As a public salutogenic resource, urban green space has been brought under the spotlight. Scholars assume that it is a typical supportive environment that can achieve both physical and psychological restoration, through emotional improvement, behavioral encouragement, social interaction, and aesthetic experience (5–13).

However, most previous studies were based on a crude comparison of natural and built environments, as well as their respective health benefits. In order to effectively guide the planning and design of urban green spaces to achieve such results, scholars have transformed abstract statistical models into theoretical correlation models of “urban green space characteristics—specific health outcome” (11, 14–20). This aims to provide a basis for empirical research on the design of green spaces from a health perspective. Therefore, numerous tools to evaluate the micro characteristics and restorative effects of the environment have been proposed and implemented (21–25). These have contributed to a more comprehensive picture of the green space and the consequent psychological benefits. The more typical and widely used is the Perceived Sensory Dimensions (PSDs) proposed by Grahn P (26) and the Perceived Restorativeness Scale (PRS) proposed by Hartig T (27). Grahn P summarized PSD as the eight different sensory experiences people get from interacting with the natural environment, including serene, nature, rich in species, space, prospect, refuge, social, and culture. On the other hand, Hartig T described PRS being the capacity of an environment to induce restorative effect through the facilitation of the feeling of fascination, being away, extent, and compatibility. The two ratings have been proven to have a distinctive relationship with different green space types. For instance, under the setting of small urban public green spaces, distinct associations are demonstrated between serene, social, nature and the PRS of people with average and high–stressed. Nature, serene, and refuge are strongly related to those in care settings. Serene, rich in species, refuge, and nature are found to be psychologically restorative in urban forests. Nature, refuge, and prospect are significantly associated with teenagers' perceived restoration in urban green spaces. This also indicates that the degree of association between PSD and PRS depends heavily on the type of green space (14, 15, 28–31) (Table 1).

**Table 1:** A review of studies in relation to the relationship of PSD and PRS.

**References**	**Region**	**Types of green space**	**Study type**	**Study aim**
Peschardt and Stigsdotter (14)	Denmark	Small public urban green spaces	Cross-sectional research	The analyses were conducted to see whether the PSDs were present or not and to determine how strong or weak they were.
Memari et al. (31)	Iran	Care setting	Controlled experiments	Identifying the relationship between environmental characteristics of restorative natural environments by determining PSDs and restorative potential.
Stigsdottera et al. (29)	Denmark	Forest	Controlled experiments	Identifying which nature qualities and spatial aspects in the Health Forest promote psychological restoration.
Chen et al. (30)	China	Forest,Park,Public Square	Cross-sectional research	Exploring how the local people perceive PSDs in relation to restoration in Chinese urban green space settings.
Akpinar (28)	Turkey	Recreational areas, Neighborhood parks, Urban park, Greenway	Cross-sectional research	Investigate the associations between the PSDs of urban green space and teenagers‘ perceived restorativeness, stress, and mental health.
Malekinezhad et al. (15)	Malaysia	Campus green space	Cross-sectional research	Identifying if the association between PSD and perceived restorativeness from a user's perspective is positive.

Yet, it is also identified that human participation is also required for users to achieve restoration among the theoretical models constructed by many scholars. Usage patterns are introduced in studies to further investigate the correlation between urban green space and recovery, such as “moving away from stressors”, “restoring capacity”, and “building capacity” (6, 19, 32–37). Specifically, as an embodiment of restorative effects, the notion of “use” mediates the process of acquiring positive restorative benefits from the environmental, be it from adjusting one's physiological or psychological condition (7, 16–18, 38–42). Furthermore, a large number of reviews also summarizes certain usage patterns where people may derive corresponding benefits from the environment, to some extent, supports the research in seeking positive link between PSD and PRS (19, 36, 43–47). For example, a lawn space can be used for both aesthetic viewing (visual stimulation) and physical activity (physiological promotion) to achieve a reduction in the expenditure of cognitive resources and a decrease in negative emotions. Therefore, as an intermediate process and bridge between the physical environment and psychological feelings, usage patterns are an important mediating variable in exploring the pathway on how PSD connected PRS.

The aforementioned empirical studies tend to focus primarily on green spaces indicated in urban land use and zoning standards, such as parks, urban forests and greenways (38, 39, 48–51). However, high-density urban development has resulted in a significant reduction in the per capita green space, leading to a request for alternative places for nature contact at non-surface level. As informal green spaces that are more closely linked to the built environment, the Public Rooftop Gardens (PRGs) have been neglected in these studies. PRG is a green open space with a certain degree of man-made facilities and natural vegetation that allows urban residents to carry out various types of activities. They are seen as an effective complement and alternative to green open spaces on the ground, especially in densely populated areas, to compensate for the lack of consideration in the provision of open space for the community (52–55). As part of the research on human- place interaction, some researchers have focused on health outcomes through establishing the relationship between the aesthetic value of planting and users' preferences for roof greening (56–61). For example, White and Gatersleben evaluated various forms of roof planting based on self-perceived restoration benefits, aesthetic value, and environmental preference. On that basis, Lee, Loder, Jungles and Nagase used plant form, height, foliage color and biodiversity as the main variables to obtain a significant relationship between users' aesthetic preferences (plant types) and their psychological comfort in rooftop space. Other researchers have experimentally controlled the restorative benefits given to observers with or without green elements in rooftop space (62–64), such as Lee, who demonstrated that green roofs provide observers with attention restoration, relief from mental fatigue and enhanced work performance through “coherence” and “fascination” in micro breaks during days.

While the two research directions confirm the restorative value of PRG from different perspectives, there are two issues where starting point of these studies overlooked. Firstly, the description of PRGs' environmental characteristics is reduced to plant aesthetics, ignoring their complex condition as green spaces. Secondly, the studies based on PRG as a view–a visually accessible window view and neglect its potential to be physically accessible and to take on more activities as open spaces. As mentioned above, the lack of research on these two fundamentals that are important influential factors to the mechanistic model, restricted the further understanding of PRG inducing restorative effect. In China, there are a large number of PRGs that can be used and experienced by all urban residents. These are informal spaces that create and renew gray areas within existing buildings, bringing open space from the public realm into the built environment (65–67). However, due to load, thermal, and usage conditions, the economic demands of stakeholders and the attribute of informal space, there is a lack of uniform standards guiding the planning and construction of PRGs (68–73), suggesting the perceived gap of comprehensive environmental conditions, i.e., PRGs have the potential to provide more public recreational activities that may lead to restorative benefits.

Thus, this manuscript aims to examine how usage patterns (UP) mediate the association of environmental characteristics (PSD) to restoration (PRS), identify the relative importance of different characteristics (PSD), usage patterns and their certain pathways linked to restoration (PRS), as well as to understand impact mechanisms of this new form of greenery using PRGs as the environmental subject. The specific objectives are to address the following questions: Q1: Is there a correlation between PSDs and PRS of PRGs? Q2: Is there a correlation between UPs and PRS of PRGs? Q3:Do UPs mediate the association between PSDs and PRS of PRGs. Q4: The certain pathways transformed from PSDs to PRS.

## Methodology and Material

### Study Context

**Figure 1:**
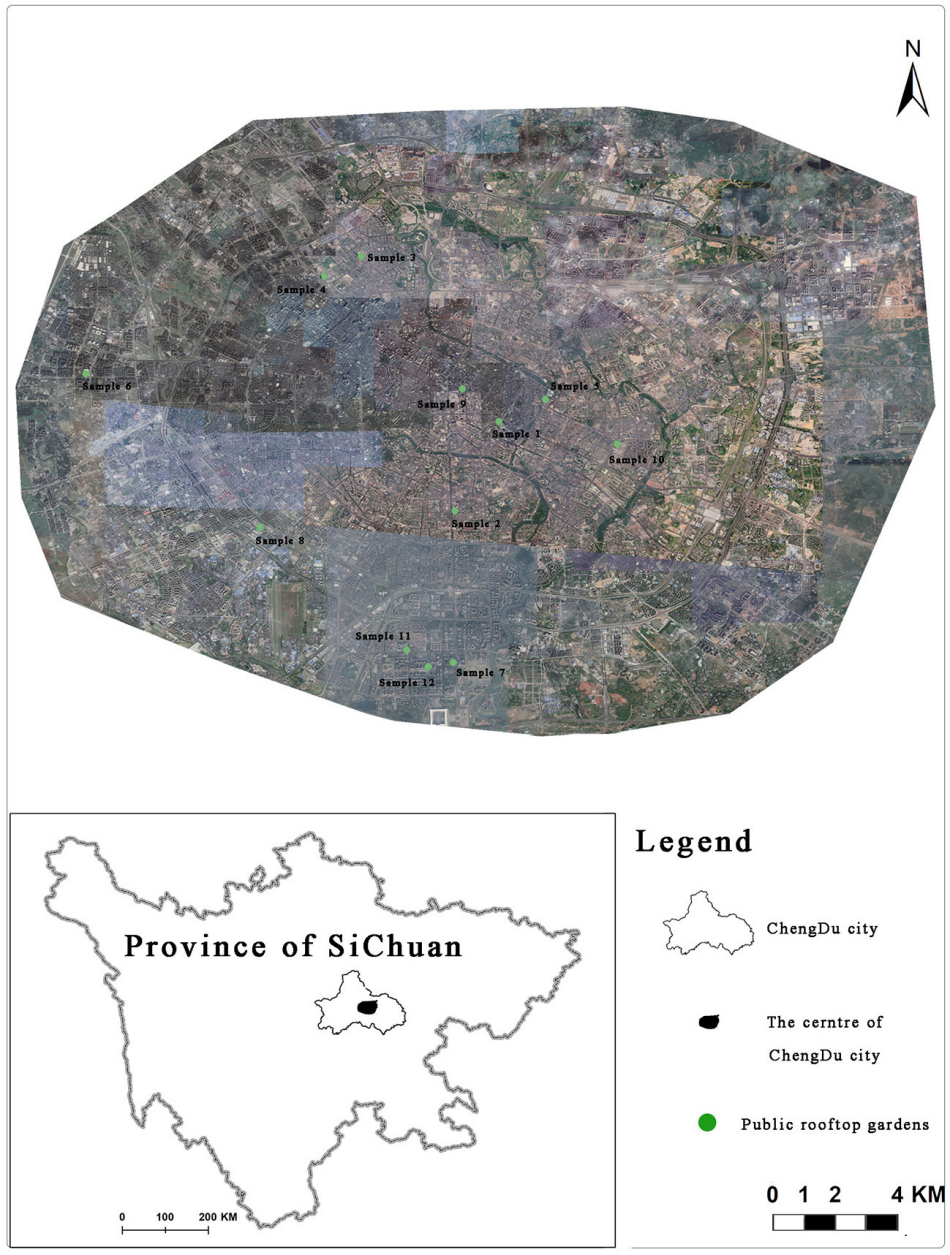
The spatial distribution of each public rooftop garden in center of ChengDu city.

Chengdu is located in the transition zone from the western Sichuan Plateau to the Sichuan Basin. It has an average temperature of 15.6~16.9°C, fewer sunny days compared to cloudy days throughout the year, and has an average annual sunshine hour of 1,003 h. The annual average precipitation is 850.9 mm, the annual average rate of evaporation is 841.1 to 1,066.1 mm, and the annual average relative humidity is 81%. All of above stated are especially suitable for the development of roof greening. Apart from climate suitability, high demolition costs and strict law enforcement on higher greening rates within a shortened timeframe foster the need to investigate the role of PRGs within the city of Chengdu. Needless to say the branding of Chengdu as a “park city” in China. These large PRGs become open green spaces for urban residents working and living in the built environment. Hence these are typical areas of study for environmental characteristics and restoration (Figure 1).

### Site Definition and Selection Criteria

PRG is also recognized as eco-roof, living roof (74), vegetated roof (69) or cool roof (75). It is defined as green space according to The National Environmental Protection Agency in the broader sense, which implies green space as “land partially or completely covered with herbs, trees, shrubs, or other vegetation” (76, 77). Hence, in this paper PRG is understood as green spaces that provide the opportunities to experience nature and increase physical activity, notwithstanding its role and capability in the regulation of urban heat island effect (78), reducing the overall energy consumption of buildings (79), rainwater detention (79), air purification (72), and increased biodiversity (78). Compared with the other urban green spaces, there was no corresponding data and information on PRGs. This is mainly because they currently do not belong to any land use typology of the city's urban planning guidelines in China. Hence, based on the pre-research and with reference to the application of PSD in traditional urban green space, there needs to be screening criteria for sample selection, in order to ensure that PSD is reasonable for this type of application. Which are shown as follows:

Roofs without enough vegetation are excluded (Green rate less than 20%).
Vacant or storage roofs are excluded.
Roofs that are not open to public are excluded.
Roofs that act like a thoroughfare (such as a podium or elevated bridge structure across two high-rise towers) are excluded.
Outdoor gardens that serve individual businesses (such as coffee shops or restaurants) are excluded.
Monotonic spaces that are unable to provide a variety of activities or host only a single activity (such as football fields and basketball courts) are excluded.

Twenty nine samples were found according to the above criteria. Then, based on information such as the area, shape, vegetation and facilities, 12 out of 29 samples were selected as the typical research samples and numbered them S1~S12. Therefore, it is safe to say the 12 PRGs selected through the screening process are: (1) green open space that meets the needs of urban residents for public access, experience and interaction, (2) green open place to hold various activities, and (3) green open space intended to serve the majority of urban residents and has a relative mix of natural vegetation and man-made facilities (Table 2).

**Table 2:** The samples of Public rooftop gardens in Chengdu (Source: All photos taken by first author unless otherwise mentioned).

**Site Number**	**Photos of sites**	**General description of sites**
Sample 1: Area:9,985.6 m^2^	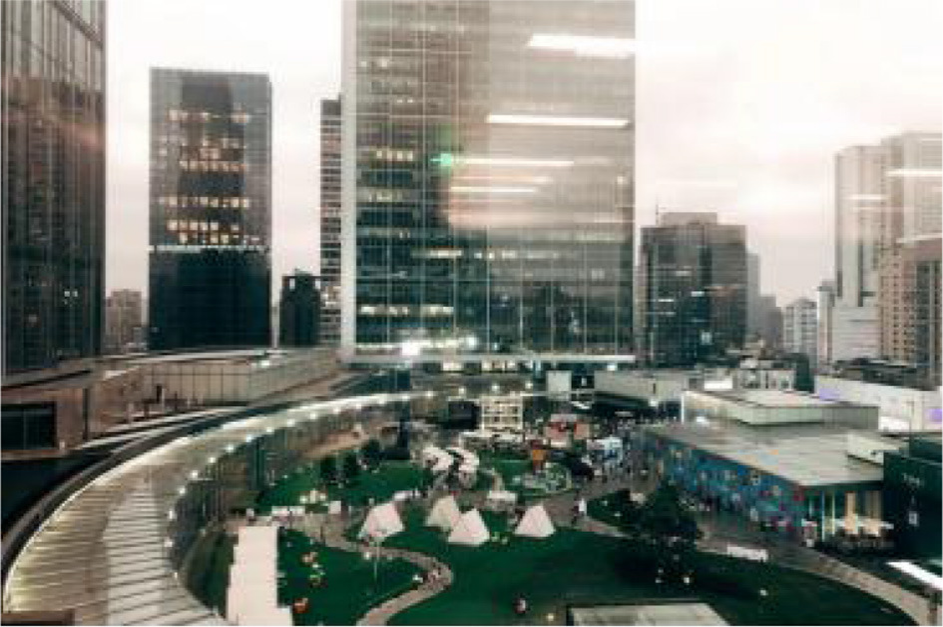	An area full of exhibitions, performances and sculptures. A wide range of recreational activities for adult and local cultural elements for people to enjoy.
		Landscape component: Lawns, trees.
		Equipment: Benches, garbage bins, exhibition facilities.
Sample 2: 8,341.5 m^2^	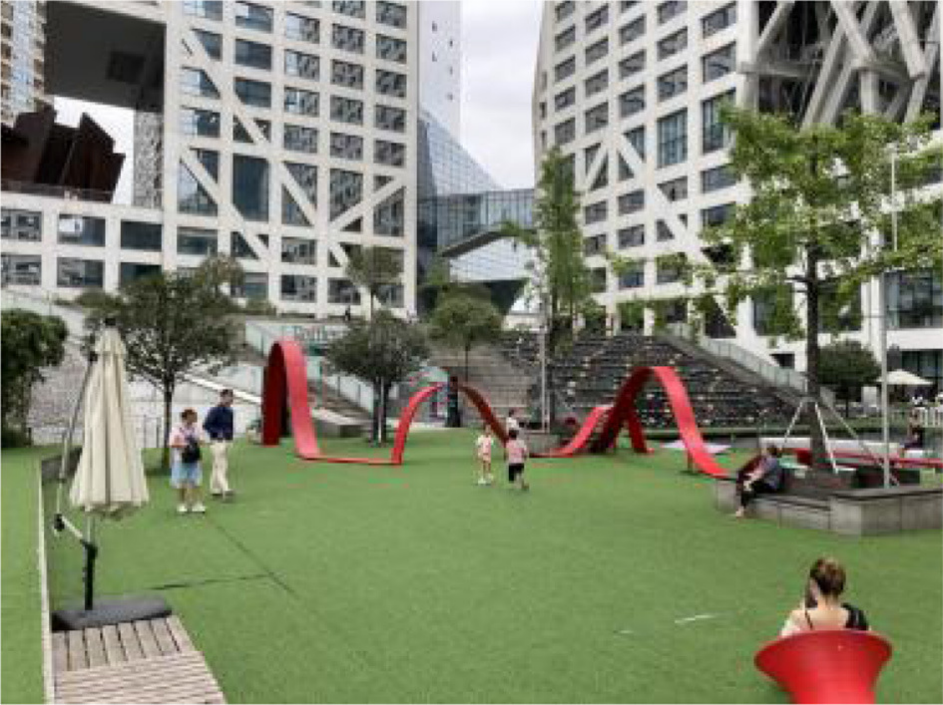	An area with cafes and a playground. Plenty of tables, chairs for socializing. while It also has many cultural symbols and water features to attract visitors.
		Landscape component: Lawns, trees, flower beds, fountains.
		Equipment: Drinking tables, sculptures, benches, garbage bins.
Sample 3: 12,980.0 m^2^	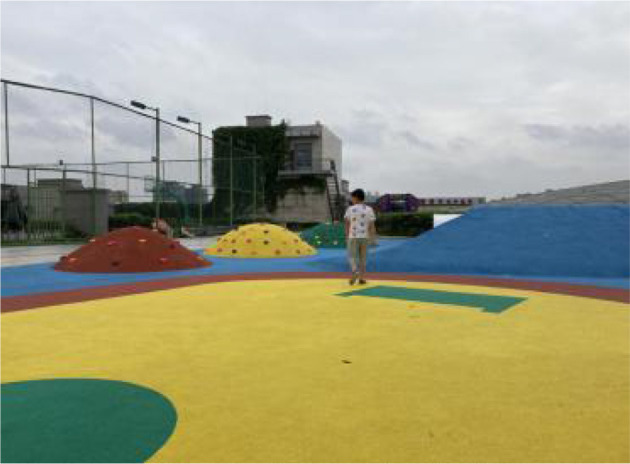	Area consisting of playgrounds, sport fields and social Spaces. A variety of Children's facilities and few trees.
		Landscape component: Lawns, shrubs, flower beds, trees, water pool.
		Equipment: Children facilities, Skateboard field, ball field, tables, shelters.
Sample 4: 9,519.0 m^2^	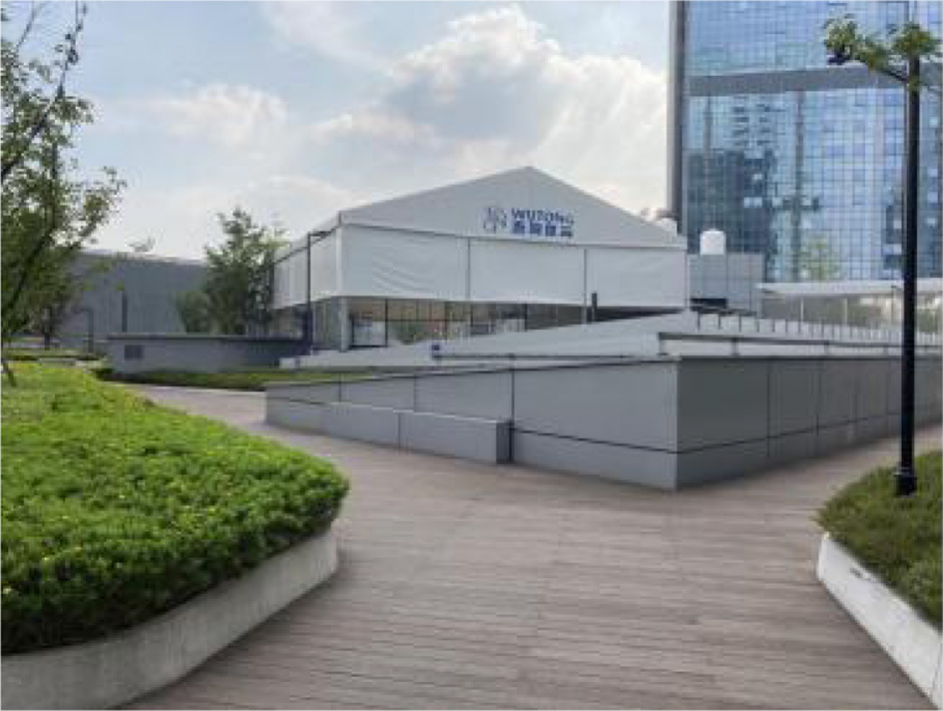	A well maintained area with an Indoor basketball court, flowerbeds, trees and many lights. Many spaces for exhibition or artist performances.
		Landscape component: Flower beds, shrubs, trees.
		Equipment: basketball field, benches, tables, fitness field.
Sample 5: 3,126.5 m^2^	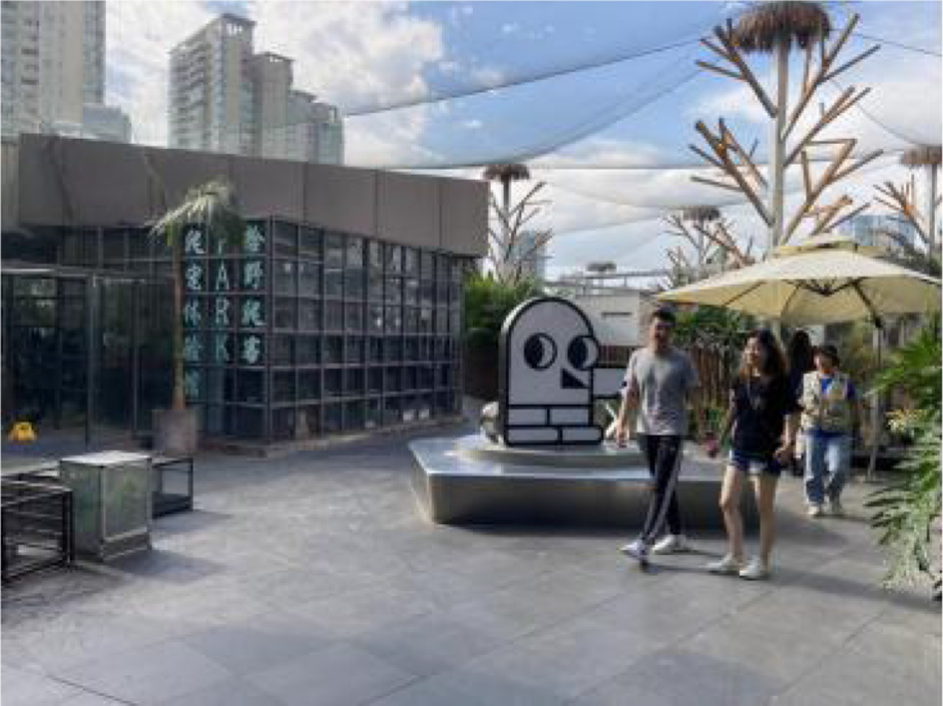	Area with several types of plants and animals, there are several tables and chairs for socializing and indoor or outdoor animal feeding or interaction space.
		Landscape component: Flower beds, shrubs, trees.
		Equipment: Drinking tables, benches, animal-feed area, horticulture area, shelters.
Sample 6: 3,323.1 m^2^	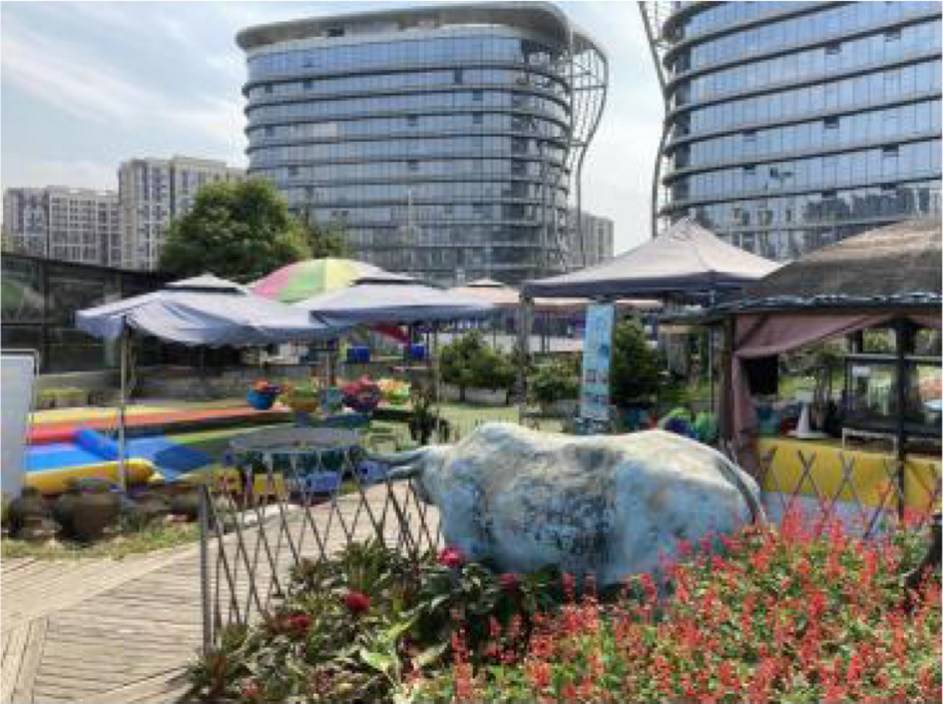	Area with many trees, bushes and hedges, there are varieties of recreational facilities for different groups and special space for Parental gardening activities with children.
		Landscape component: Lawn, flower beds, shrubs, trees.
		Equipment: Children facilities, benches, horticulture area, animal-feed field, garbage bins, shelters.
Sample 7: 4,606.6 m^2^	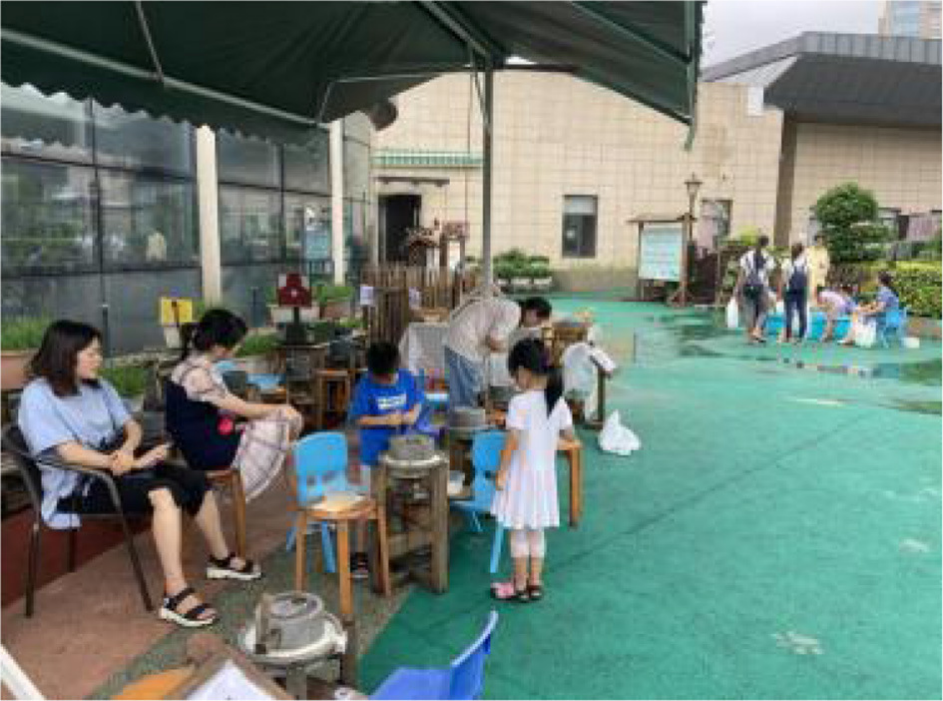	Area with horticultural or gardening space for family, there are also educational space for sports or outdoor environmental knowledge learning.
		Landscape component: Flower beds, shrubs, trees.
		Equipment: Benches, tables, animal-feed area, horticulture area, garbage bins, shelters.
Sample 8: 18,555.0 m^2^	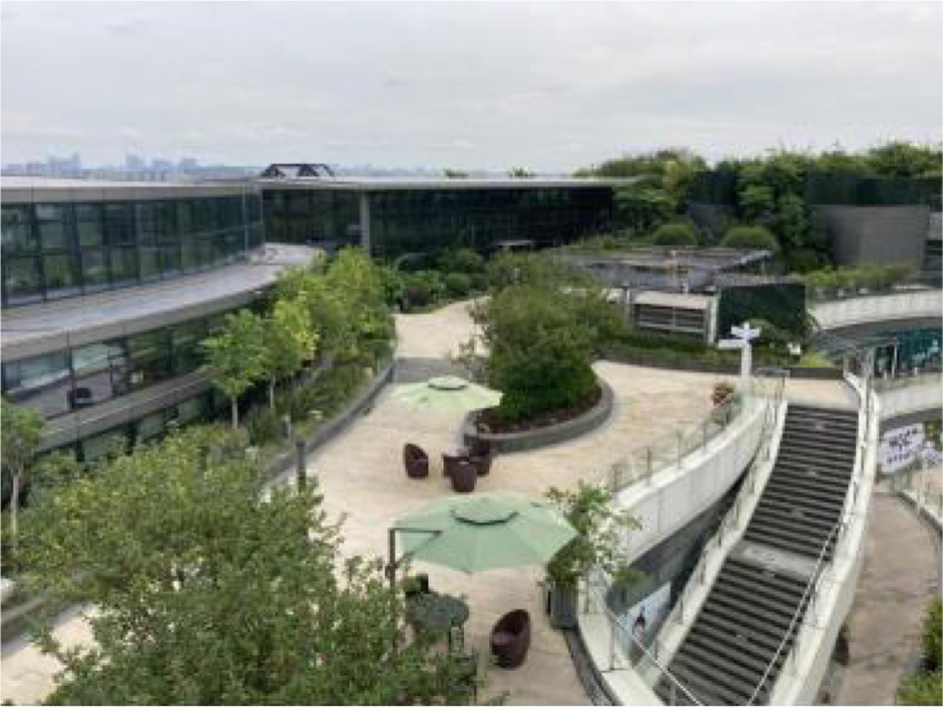	Area surrounded by many trees bushes and free growing lawns. Different types of plant combinations creates impressive space for users to enjoy. People can have social interaction or have space to be alone.
		Landscape component: Lawn, flower beds, shrubs, trees, water elements.
		Equipment: Benches, garbage bins, tables, fitness field, children facilities, shelters.
Sample 9: 6,370.40 m^2^	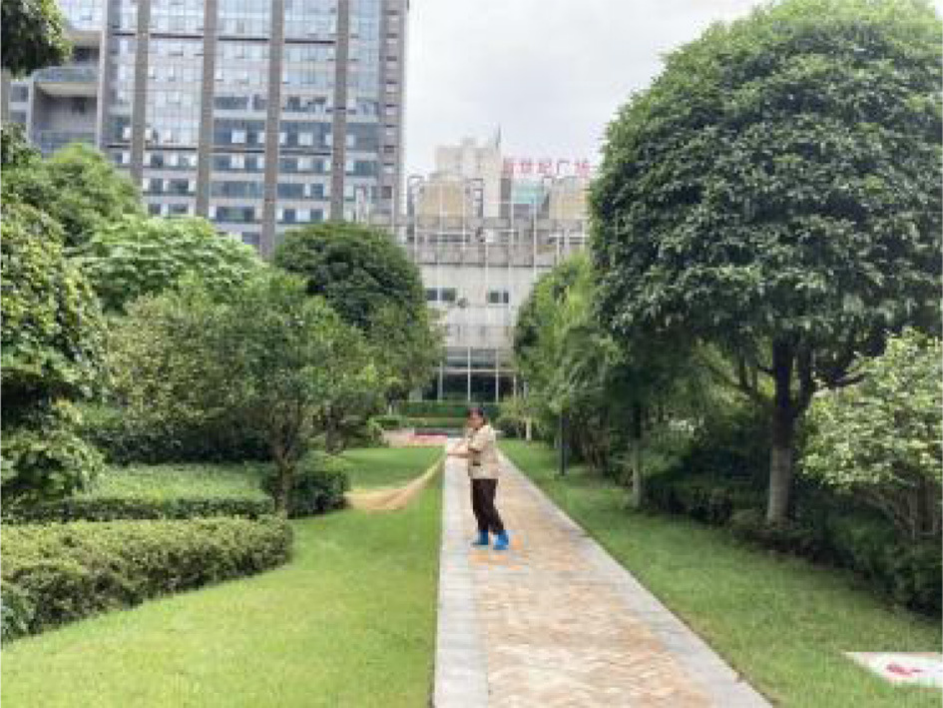	A well maintained area with trees, hedges, growing lawns and flowerbeds. Large trees create semi-enclosed atmosphere. Many benches provide residents with break space, where people can enjoy greenery view.
		Landscape component: Lawn, trees, shrubs, flower beds.
		Equipment: benches, garbage bins, tables, fitness field, children facilities.
Sample 10: 8,379.0 m^2^	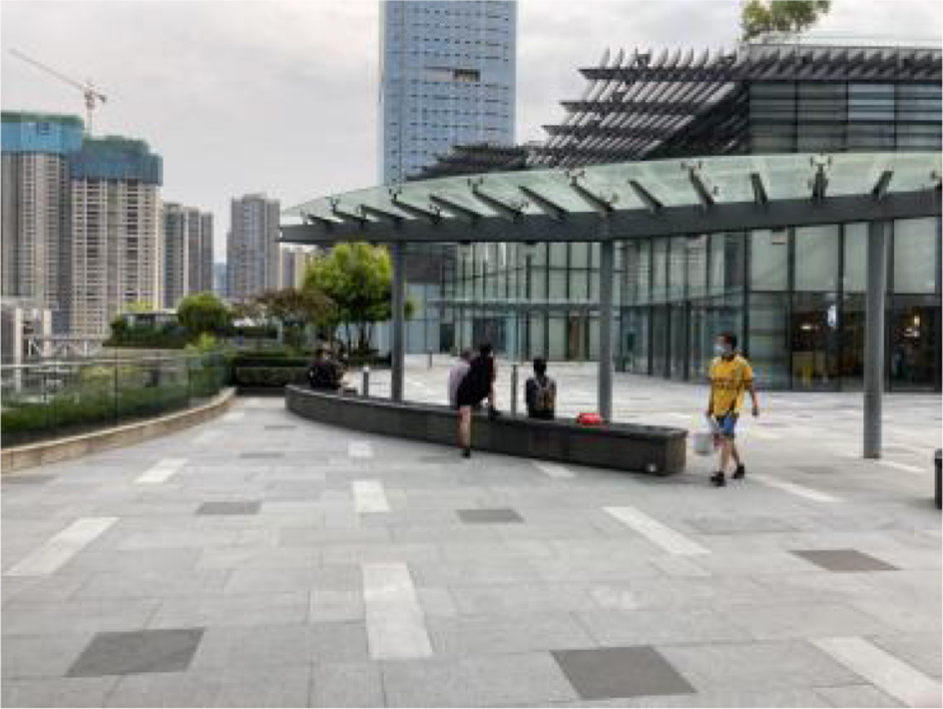	An area dominated by short-stay facilities and hedges. There are several benches where people can sit and look at the up-to-date art performances.
		Landscape component: Trees, shrubs, flower beds.
		Equipment: benches, garbage bins, shelter.
Sample 11: 3,454.1 m^2^	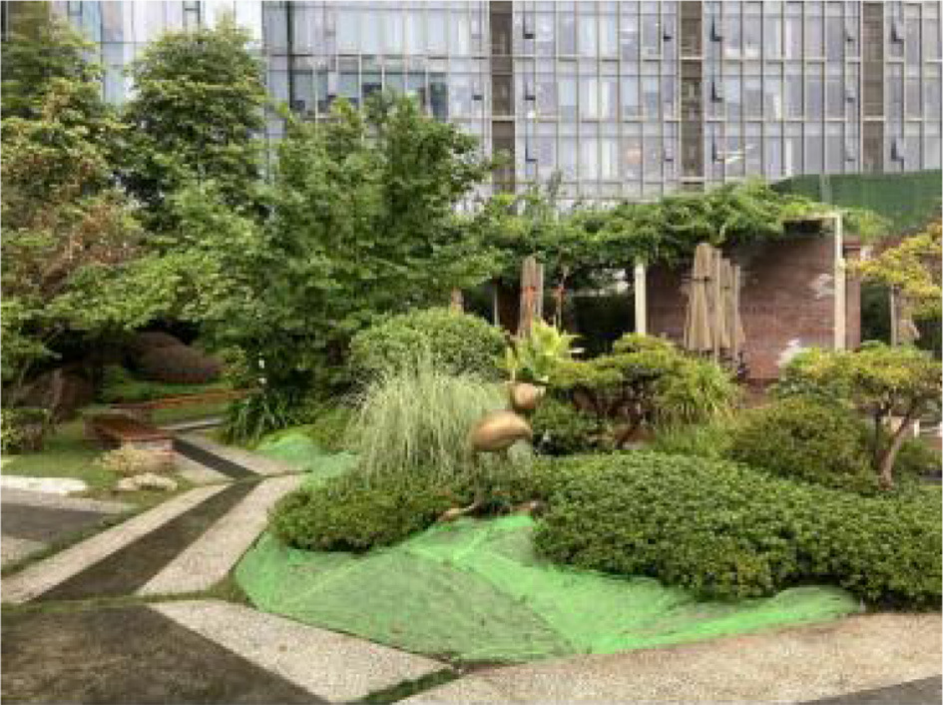	Area surrounded by many trees bushes and lawns. A social environment with tables and benches for people to drink or chat.
		Landscape component: Lawn, trees, shrubs, flower beds, water elements.
		Equipment: benches, garbage bins, shelter, drinking tables, shelters.
Sample 12: 3,975.7 m^2^	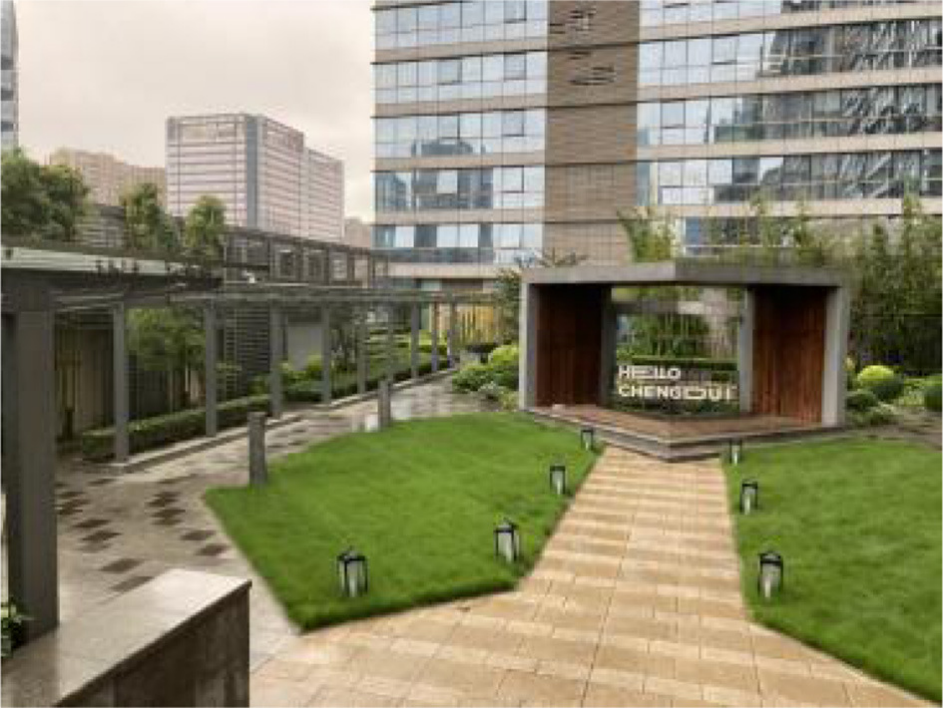	Area with some lawns, bushes and tables. Many workers feels Silent, have lunch break or seek inspiration here.
		Landscape component: Lawn, trees, shrubs, flower beds.
		Equipment: benches, shelters, drinking tables, garbage bins.

### Data Collection

The evaluation of environmental characteristics, usage pattern and restorative effect of PRGs in Chengdu were measured by means of an onsite questionnaire survey. We revised and tested ratings based on the actual condition of sites during the pre-research, then conducted the face to face interviews in the formal questionnaire distribution.

In the Pre-research started on 5 August 2020, every item of the PSDs was rated against actual settings of 12 PRGs on-site by three professional landscape architects. Due to the fact that most items associated with PSDs outlined in previous studies were based off traditional urban green environment, such as small urban green spaces, forests, parks, or care settings, it meant that some of the specific characteristics which described each PSD could not be translated directly into PRGs due to contextual differences. We therefore screen out the items considered not relevant when evaluating PRGs. Meanwhile, real-time status of the users is also being recorded to provide an indication and summary of their usage patterns, i.e., animal feeding, parent-child interaction, meditation and rest, sports and physical exercise, or looking for inspiration.

As a follow up, formal questionnaire distribution started on 31 September 2020, and the frequency of visits to the 12 sites was controlled to an average of six visits per month, which were conducted until the end of the month on 31 June 2021. During this period, 48 visits were conducted by 1–2 members per site sample (at different times of the month, in the morning, around noon, in the evening and on weekends, for 1–2 h each time to reach as many different users as possible). Each survey was distributed at 12 sites at the same time to ensure that external environmental influences such as weather were consistent. Respondents were randomly selected amongst the visitors of the selected PRGs. Questions were asked before distribution to ascertain whether the respondents who agreed to be interviewed were local residents (or lived in the area more than 1 year) to ensure that the content of the questionnaire was a comprehensive reflection of the most realistic current use of the site each time. Those willing to participate were then asked to fill out a questionnaire during their stay in the area, based on their overall perception of the site and their willingness to carry out the corresponding activities within 6 months (Supplementary Table S1).

The questionnaire consisted of five sections. The first part was a survey on motivation, frequency, background, and relevant demographic characteristics (gender, age, education and income level, presence of preschool children, insider or visitor). The second part was an evaluation of the eight perceived dimensions in PRGs. The third part was a questionnaire based on the activities (usage patterns) of the respondents in the specific areas of the site, and the fourth part was a questionnaire on the perceived restorativeness of visiting the site (The fifth part is not relevant in this paper). Correspondingly, a 7-degrees Likert scale was used for the evaluation. Higher scores indicate a greater willingness or agreement of specific item of the questionnaires. The scores of negative questions were being revised for consistency.

During the site investigation, 17,183 users of the sites were recorded, and 1,376 questionnaires (211 paper and 1,165 electronic questionnaires) were distributed. Out of all distributed questionnaires, 899 questionnaires were recovered with a questionnaire recovery rate of 63%. Of these recovered questionnaires 876 were valid, meaning that 97% of the recovered questionnaires were usable. The recovered questionnaires provided an indication to the demographic of all the PRGs users: External visitors (73.3%), females (53%), people with preschool children in family (54.8%), young and middle-aged people (18–39 of age, 58.5%), people with an average lower income (<4,999, 59.2%), and people with a lower education (under vocational education, 70.2%) constitute the main use groups of the site. However, it is utmost important to note that no significant differences were found between the responses of people with different background subjects.

### Data Screening and Analysis

Structural Equation Model (SEM) was chosen to construct the final theoretical model in this paper because it (1) introduces latent variables that can be included in the evaluation of multiple specific items in a complex, multi-linked statistic, facilitating the presentation of the causal structure of the variables from a holistic perspective;(2) SEM is similar to multiple regression and path analysis in that they are solved using a system of coupled equations, but unlike the two, it allows for measurement error between variables; (3) SEM can eliminate confounding factors while taking into account the relationship between multiple variables.

The modeling steps were shown in turn: comprehensive data validation tools were used upfront, followed by series of linear regression analysis to screen out uncorrelated variables in order to simplify the construction of structural equation model. Finally, the relevant variables were input into AMOS to establish SEM, and the path coefficients were used to determine whether the theoretical conjecture was valid and the respective contribution.

Specifically, original data was first statistically analyzed to ensure whether it was available for subsequent statistical analysis. Mean and standard deviation were used to describe the data for continuous variables, while frequency and percentage were used to define categorical variables. Then, Harman's one-way ANOVA was used as the test for common method bias to eliminate artificial covariation of the evaluation ratings that can seriously confound results and potentially mislead conclusions.

After that, exploratory factor analysis (EFA), confirmatory factor analysis (CFA) and Cronbach's alpha were used in this study to verify if the data supported the grouping of dimensions of different ratings. Furthermore, corresponding mathematical structures were tested using CR (composite reliability) and AVE (average variance extracted), respectively.

Then a series of multiple linear regression analysis was then used to determine the possible association among the independent variables (PSD ratings), mediating variables (UP ratings) and dependent variables (PRS ratings). Specifically, the models estimate which independent and mediating variables have significant effects on dependent variables, and this is to simplify post-modeling by filtering out irrelevant variables.

All of the above are prerequisite test for the development of a valid mathematical model. Finally, an optimized structural equation model of latent variables was developed to verify the mediating role of each mediating variable between the independent variable and the dependent variable through AMOS. The test level (Significant level) was 0.05, i.e., *P* < 0.05 indicated that the difference was statistically significant. The model reveals what degree of environmental characteristics are associated with users' restorative effects and the mediating role of usage patterns through path coefficients. It also illustrates the different pathways between certain environmental characteristics and restorative effects.

## Results

Results from these analyses are described and presented in the following sections: Confirmatory analysis and Main analysis. These sections follow the steps of Data screening and analysis corresponding to the research questions highlighted in the previous chapter. Confirmatory analysis aims to verify whether the data supported the grouping of dimensions of different ratings for the subsequent analysis, followed by Main analysis to respond the research question.

### Confirmatory Analysis

Prior to answering the research questions, it was firstly verified whether the variables supported the grouping of dimensions described as PSD as well as PRS, while the dimensionality of the data related to UP was examined (Tables 3–5).

**Table 3:** Corrected correlations between PSDs items and PSDs.

**Item**	**Component**								**Communalities**
	**Space**	**Social**	**Culture**	**prospect**	**refuge**	**Serene**	**Nature**	**Rich in species**	
**Space (latent variables)**									
Spacious	0.792								0.705
Areas not crossed by paths	0.791								0.670
Lots of trees	0.777								0.645
Places sheltered from the wind	0.776								0.677
Places where people can gather	0.790								0.687
**Cronbach's alpha** **=** **0.878**									
**Social (latent variables)**									
Entertainment		0.801							0.683
Exhibitions		0.778							0.654
Paths with hard surfaces		0.807							0.709
General good lighting		0.795							0.686
Plenty of people		0.729							0.615
**Cronbach's alpha** **=** **0.872**									
**Culture (latent variables)**									
Fountains			0.815						0.710
Statues			0.799						0.698
Foreign plants			0.820						0.710
Flowers			0.853						0.782
**Cronbach's alpha** **=** **0.871**									
**Prospect (latent variables)**									
Plane, well-cut grass				0.842					0.732
Prospect				0.836					0.718
Cut lawns				0.849					0.750
Small ball grounds				0.758					0.615
**Cronbach's alpha=0.852**									
**refuge(Latent variables)**									
Many bushes					0.753				0.614
Tables and benches					0.789				0.667
Play equipment					0.763				0.649
Watching people being active					0.784				0.675
**Cronbach's alpha** **=** **0.820**									
**Serene (latent variables)**									
Silent and calm						0.751			0.610
Clean and well maintained						0.735			0.601
Not crowded						0.783			0.657
Feel safe						0.765			0.650
**Cronbach's alpha** **=** **0.797**									
**Nature (latent variables)**									
Nature quality							0.854		0.794
Free growing lawns							0.839		0.773
Wild and untouched							0.859		0.810
**Cronbach's alpha** **=** **0.869**									
**Rich in species (latent variables)**									
Natural animals. plant and animal populations								0.871	0.825
Many native plants to study								0.880	0.828
**Cronbach's alpha** **=** **0.797**									
Eigenvalue	3.388	3.359	2.894	2.816	2.625	2.524	2.347	1.645	
% of variance	10.930	10.834	9.335	9.083	8.467	8.142	7.571	5.306	
Cumulative %	10.930	21.764	31.099	40.182	48.650	56.792	64.363	69.669	

**Table 4:** Corrected correlations between UPs items and UPs.

**Item**	**Component**						**Communalities**
	**Retreat**	**Interpersonal**	**Family-bonding**	**Exercise**	**Sightseeing**	**Nature touching**	
**Retreat(Latent variables)**							
Strolling	0.855						0.763
Reading	0.831						0.748
Meditation	0.840						0.765
Inspiration	0.862						0.794
Lunch break	0.846						0.765
**Cronbach's alpha** **=** **0.924**							
**Interpersonal (latent variables)**							
Gathering for a meal		0.881					0.795
Playing cards and chess		0.843					0.726
Chat with Friends		0.867					0.768
Dating		0.866					0.761
Square dancing		0.869					0.774
**Cronbach's alpha** **=** **0.923**							
**Family-bonding (latent variables)**							
Feed animals			0.848				0.771
Play with children-facility			0.843				0.756
Environmental learning			0.838				0.750
Horticultural work			0.859				0.782
**Cronbach's alpha=0.897**							
**Exercise(Latent variables)**							
Running				0.872			0.782
Ball games				0.897			0.822
Equipment fitness				0.855			0.758
**Cronbach's alpha** **=** **0.862**							
**Sightseeing (latent variables)**							
Participation in site activities					0.850		0.768
Photo-op landmark scenery					0.855		0.775
Appreciation of artificial landscape					0.849		0.761
**Cronbach's alpha** **=** **0.849**							
**Nature touching (latent variables)**							
Breathing fresh air						0.835	0.748
Appreciation of the greenery						0.796	0.679
Enjoy the outdoor weather						0.808	0.705
**Cronbach's alpha** **=** **0.791**							
Eigenvalue	3.846	3.836	3.058	2.356	2.303	2.117	
% of variance	16.724	16.677	13.296	10.245	10.013	9.206	
Cumulative %	16.724	33.400	46.696	56.941	66.954	76.160	

**Table 5:** Corrected correlations between PRS items and PRS.

**Item**	**Component**				**Communalities**
	**Compatibility**	**Fascination**	**Coherence**	**Being away**	
**Compatibility (latent variables)**					
I can do things I like here	0.736				0.672
I have a sense that I belong here.	0.707				0.633
I have a sense of oneness with this setting.	0.722				0.648
Being here suits my personality.	0.783				0.752
I could find ways to enjoy myself in a place like this	0.784				0.728
**Cronbach's alpha** **=** **0.876**					
**Fascination (latent variables)**					
The setting has fascinating qualities		0.754			0.668
My attention is drawn to many interesting things		0.649			0.610
I would like to get to know this place better		0.682			0.673
There is much to explore and discover here		0.669			0.579
I would like to spend more time looking at the surroundings		0.748			0.648
**Cronbach's alpha** **=** **0.853**					
**Coherence (latent variables)**					
There is too much going on.			0.680		0.593
It is a confusing place.			0.753		0.689
There is a great deal of distraction			0.801		0.776
It is chaotic here.			0.829		0.784
**Cronbach's alpha** **=** **0.866**					
**Being away (latent variables)**					
It is an escape experience				0.846	0.871
It gives me a good break from day-to-day routine				0.817	0.860
**Cronbach's alpha** **=** **0.840**					
Eigenvalue	3.325	3.105	3.048	1.707	
% of variance	20.782	19.404	19.048	10.666	
Cumulative %	20.782	40.187	59.234	69.901	

Specifically, the results showed that the cumulative variance contribution of the PSD rating was 69.669% > 60%, i.e., when eight common factors (refuge, social, serene, space, nature, rich in species and culture) were extracted. While the cumulative variance contribution of the UP rating was 76.160% > 60%, i.e., When six common factors (retreat, exercise, interpersonal, family-bonding, nature touching, sightseeing) were extracted. Finally, the cumulative variance contribution of the PRS rating was 69.901% > 60%, i.e., When four common factors (fascination, compatibility, coherence and being away) were extracted. That is, the three can better cover the subject of the study through a principal component analysis strategy.

### Main Analysis

#### Q1: The Correlation Between PSDs and PRS?

The effect of the PSD rating (as the X-value of the equation) on the PRS rating (Y-value) was explored by multiple linear regression analysis, incorporating variables such as nature, space, culture, social, refuge, serene, prospect, rich in species and perceived restorativeness as the dependent variable. The regression results show that culture, prospect and rich in species did not have significant effects on the restoration (*P* > 0.05). Nature, space, social, refuge and serene have significant effects on the restoration (*P* < 0.001), and the β values were 0.158, 0151, 0.221, 0.154, 0.186, and 0.022, respectively, so it was concluded that these dimensions had significant positive effects on Y (Table 6).

**Table 6:** The regression model of PSDs on PRS.

**Perceived sensory dimensions**	**B**	**Std. Error**	**Beta**	**t**	**P**	**Tolerance**	**VIF**
(Constant)	2.852	0.090		31.570	0.000		
**Nature**	0.074	0.015	0.158	5.016	**0.000**	0.808	1.238
**Space**	0.079	0.017	0.151	4.604	**0.000**	0.739	1.353
Culture	−0.014	0.016	−0.027	−0.865	0.387	0.803	1.246
**Social**	0.110	0.016	0.221	6.683	**0.000**	0.732	1.366
**Refuge**	0.077	0.016	0.154	4.806	**0.000**	0.779	1.283
**Serene**	0.091	0.016	0.176	5.532	**0.000**	0.788	1.270
prospect	−0.005	0.016	−0.009	−0.298	0.766	0.886	1.129
Rich in Species	0.010	0.014	0.022	0.732	0.465	0.865	1.156

*The highlighted bold factors represent significantly correlated variables*.

#### Q2: The Correlation Between UPs and PRS?

The effect of the UP rating (as the M-value of the equation) on the PRS rating (Y-value) was explored by multiple linear regression analysis, incorporating variables such as exercise, retreat, interpersonal, family-bonding, nature touching, sightseeing and perceived restorativeness as the dependent variable. The regression results show that exercise and sightseeing did not have significant effects on the restoration (*P* > 0.05). Retreat, interpersonal, family-bonding, nature touching have significant effects on the restoration (*P* < 0.001), and the β values were 0.210, 0.216, 0.214 and 0.174, respectively, so it was concluded that these dimensions had significant positive effects on Y (Table 7).

**Table 7:** The regression model of UPs on PRS.

**Usage patterns**	**B**	**Std. Error**	**Beta**	**t**	**P**	**Tolerance**	**VIF**
(Constant)	3.037	0.077		39.315	0.000		
Exercise	0.003	0.015	0.005	0.168	0.867	0.929	1.076
**Retreat**	0.083	0.013	0.210	6.361	0.000	0.751	1.332
**Interpersonal**	0.088	0.012	0.216	7.210	0.000	0.920	1.087
**Family-bonding**	0.087	0.013	0.214	6.749	0.000	0.816	1.226
**Nature touching**	0.087	0.016	0.174	5.560	0.000	0.840	1.190
Sightseeing	0.006	0.014	0.013	0.434	0.664	0.859	1.165

*The highlighted bold factors represent significantly correlated variables*.

#### Q3: The Mediating Effect of UPs

Based on the results of the aforementioned regression analysis, a preliminary structural equation model of UP ratings between the PSD and the PRS ratings was also constructed after removing the irrelevant variables, incorporating nature, space, social, refuge and serene as independent variables. Incorporating retreat, interpersonal experience, family-bonding activities and nature touching as mediating variables. Incorporating restoration as the dependent variable. The model plots and analysis results are presented below.

The results of the structural equation model analysis are presented in the table. The analysis results show that: the effect of nature on family-bonding activities is not significant (*P* = 0.096 > 0.05), social has a non-significant effect on retreat (*P* = 0.945 > 0.05), it also has a non-significant effect on nature touching (*P* = 0.674 > 0.05), and refuge had a non-significant effect on nature touching (*P* = 0.505 > 0.05), serene had a non-significant effect on interpersonal experience (*P* = 0.157 > 0.05), serene had a non-significant effect on Interpersonal experience (*P* = 470.05 > 0.05). This means that 14 of the 20 pathways are significantly correlated, which prove the mediating role of usage pattern between PSD and PRS (Table 8).

**Table 8:** Preliminary results of structural equation modeling analysis.

**Pathway**			**Standard**	**Unstandard**	**SE**	**t**	**P**
**Retreat**	**←**	**Nature**	0.178	0.191	0.039	4.930	***
**Interpersonal**	**←**	**Nature**	0.126	0.141	0.040	3.515	***
Family-bonding	**←**	Nature	0.061	0.065	0.039	1.663	0.096
**Nature touching**	**←**	**Nature**	0.248	0.215	0.034	6.249	***
**Retreat**	**←**	**Space**	0.189	0.230	0.044	5.211	***
**Interpersonal**	**←**	**Space**	0.220	0.281	0.046	6.057	***
**Family-bonding**	**←**	**Space**	0.205	0.248	0.045	5.531	***
**Nature touching**	**←**	**Space**	0.174	0.171	0.039	4.440	***
Retreat	**←**	Social	0.002	0.003	0.044	0.069	0.945
**Interpersonal**	**←**	**Social**	0.189	0.245	0.047	5.188	***
**Family-bonding**	**←**	**Social**	0.152	0.187	0.046	4.111	***
Nature touching	**←**	Social	0.016	0.016	0.039	0.421	0.674
**Retreat**	**←**	**Refuge**	0.132	0.150	0.042	3.550	***
**Interpersonal**	**←**	**Refuge**	0.169	0.201	0.044	4.534	***
**Family-bonding**	**←**	**Refuge**	0.237	0.267	0.044	6.126	***
Nature touching	**←**	Refuge	0.027	0.024	0.037	0.666	0.505
**Retreat**	**←**	**Serene**	0.225	0.280	0.048	5.834	***
Interpersonal	**←**	Serene	−0.053	−0.069	0.049	−1.416	0.157
Family-bonding	**←**	Serene	0.027	0.034	0.047	0.719	0.472
**Nature touching**	**←**	**Serene**	0.210	0.211	0.042	5.046	***
Restoration	**←**	Serene	0.175	0.073	0.017	4.326	***
Restoration	**←**	Refuge	0.133	0.051	0.015	3.402	***
Restoration	**←**	Social	0.221	0.092	0.016	5.801	***
Restoration	**←**	Space	0.078	0.032	0.016	2.003	0.045
Restoration	**←**	Nature	0.112	0.040	0.014	2.933	0.003
Restoration	**←**	Nature touching	0.140	0.058	0.017	3.385	***
Restoration	**←**	Family-bonding	0.181	0.061	0.013	4.687	***
Restoration	**←**	Interpersonal	0.138	0.044	0.012	3.660	***
Restoration	**←**	Retreat	0.147	0.050	0.013	3.877	***

The symbol *** means p < 0.001. The highlighted bold factors represent significantly correlated pathways of variables.

#### Q4: The Pathway of PSDs Linked to PRS of PRG

**Figure 2:**
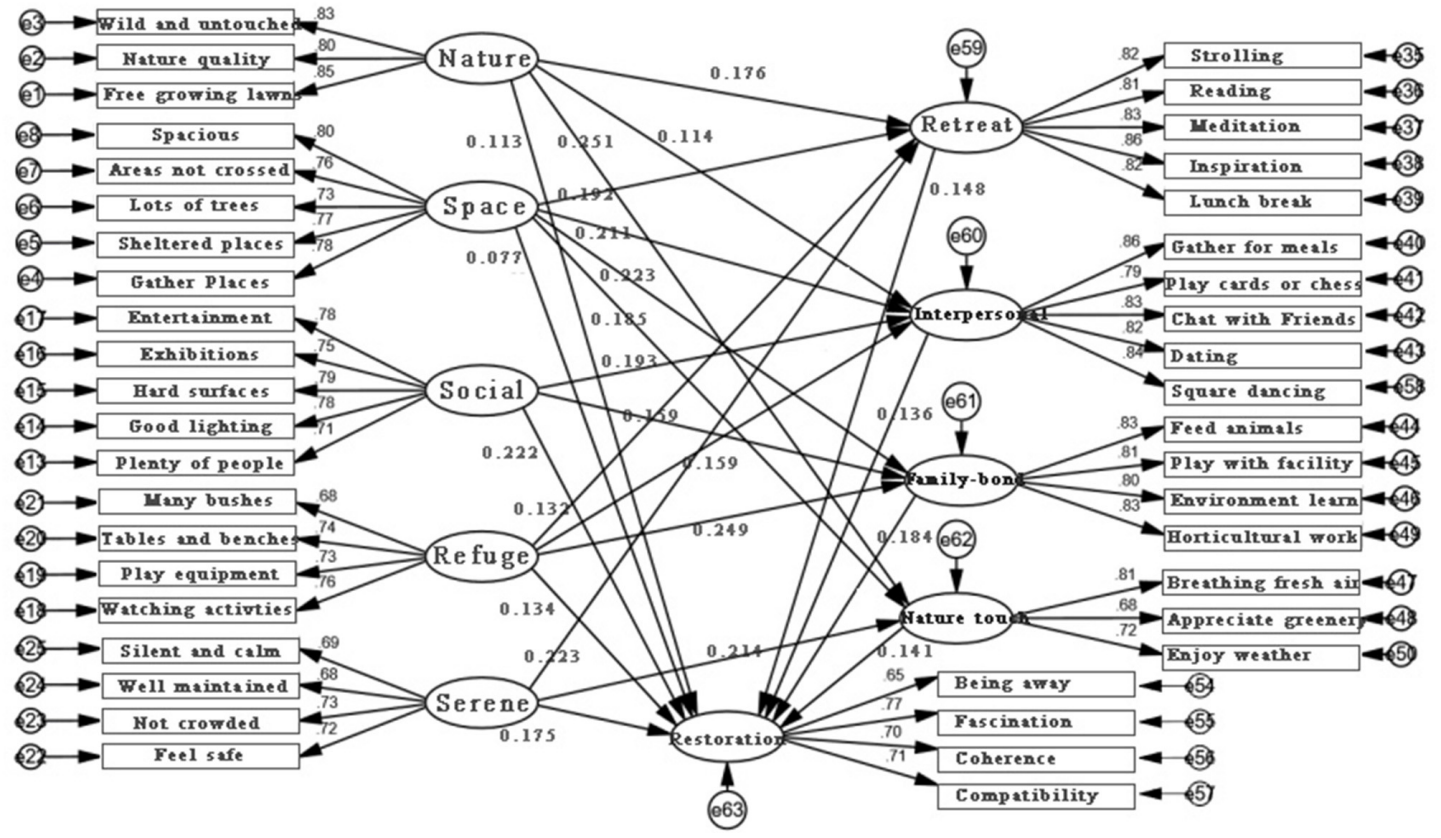
Final result of structural equation modeling analysis.

After optimization of the structural equation model by eliminating several insignificant paths between X—M—Y variables, fit indices of the optimized model all met the fit criteria, and it was considered to be a good model structure that was supported by the data. The results of the optimization of the non-significant paths in the structural equation model, as previously mentioned, are presented below (Figure 2).

The results of the optimized structural equation model show that for the effect of environmental characteristics on restorative effects, nature, space, social, refuge and serene all show a significant positive effect on restoration (*p* < 0.05), with standardized path coefficients of 0.113, 0.077, 0.222, 0.134 and 0.175, respectively. The effect of the mediating variable M on the dependent variable Y was as follows: retreat, interpersonal experience, family-bonding activities and nature touching all have a significant positive effect on restoration (all *p* < 0.001), with standardized path coefficients of 0.148, 0.136, 0.184, and 0.141, respectively.

To verify the different pathways between environmental characteristics and restorative effects, each path coefficients of independent variable X on mediating variable M will be covered in detail (Table 7). For example, the dimension of nature achieved restoration through retreat, interpersonal experience and nature touching (all *p* < 0.01), with standardized path coefficients of 0.176, 0.114 and 0.251, respectively. The dimension of space achieved restoration through family-bonding activities, interpersonal experience, retreat, and nature touching (all *p* < 0.001), with standardized path coefficients of 0.192, 0.211, 0.223, 0.185, respectively. The social dimension achieved restoration through interpersonal experience and family-bonding activities (*p* < 0.001), with standardized path coefficients of 0.193 and 0.159, respectively. The dimension of refuge achieved restoration through family-bonding activities, interpersonal experience and retreat. (*p* < 0.001), with standardized path coefficients of 0.132, 0.159 and 0.249, respectively. The dimension of serene achieved restoration through retreat and nature touching (*p* < 0.001). The standardized path coefficients were 0.223 and 0.214, respectively.

## Discussion

### The Relationship Between PSD, UP and PRS on PRG

One of the main research questions was to examine how through different use, certain environmental characteristic could provide restorative benefits. Our results confirmed the hypothesis that out of the 20 theoretical pathways constructed by the PSD (x)—UP (m)—PRS (y), only 14 formed a significant correlation. This suggests that environmental characteristics facilitate some specific usage patterns in a way that allows the user to achieve recovery (Table 9).

**Table 9:** Pathways corresponding to each PSD linked PRS.

**Perceived sensory dimensions**	**Usage pattern**
Nature	Retreat
	Interpersonal experience
	Nature touching
Space	Family-bonding activities
	Interpersonal experience
	Retreat
	nature touching
Social	Interpersonal experience Family-bonding activities
Refuge	Family-bonding activities
	Interpersonal experience
	Retreat
Serene	Retreat
	Nature touching

In response to the first research question, it was found that there is a positive relationship between PSDs and PRS. Social and serene are repeatedly the most important PSDs connected to PRS. This result correlates with the conclusion outlined in the research on small public urban green spaces (SPUGS) (14). This is considerably different when we look at PSD and the respective PRS in different settings. For example, refuge, rich in species are the important PSDs associated with urban forest (29); nature and serene within care settings (31); as well as refuge and prospect under park setting (28). The findings of this paper may be attributed to the following reasons: in comparison to the European, Chinese residents tend to socialize in an outdoor environment for stress relief rather than in the hustle and bustle of urban life (30, 80). PRGs can hence potentially compensate for basic needs of socializing in natural environments on busy workdays within urban areas. Similarly, compared with SPUGS, PRGs are elevated that they are far away from the source of vehicular noise on the ground, which could be seen as a scarce green space for people to obtain tranquility or peace in the built environment (62). Therefore, PRGs are easily perceived as places for temporary escape from the pressure of life and work. It is worth mentioning that the above two dimensions seem to contradict when placed within an environment. However, it may be due to the relatively large scale of PRGs, PRGs design strategies that improve delineation as well as management through a well- established operation and maintenance regime, which ensure the integration and coexistence of these two (59, 65, 70, 73).

Regarding to second research question, there is a correlation between UPs and PRS according to the result. Retreat and family-bonding activities are repeatedly most important mediating variables linked PSDs and PRS of PRG. It coincides with some scholars underpinnings on restorative experience of PRG (52, 54, 55, 62, 81). For instances, to take the children out, to find peace and quietness. Unexpectedly, out of all UPs, nature touching had relatively low impact on the restorative effect. As a contrary to green spaces on ground whereby the most restorative experience would normally be nature contact (32, 82–89), this study confirms that the current restorative function of PRG was possibly closer to being a participatory farm or place for short-term break, than being a natural space, from the perspective of users.

On the final research question, social, refuge, serene, space and nature were inducted into restorative effects through four patterns of use: retreat, nature touch, interpersonal interaction and family-bonding activities. Our findings validate the importance of mediating mechanisms in restorative environments, which supported previous study conclusions (32–35). Specifically, this study revealed that environmental characteristics of nature such as “wild and untouched” and “growing lawns” contribute more to the creation of a space where visitors feel the inherent power of the environment without human intervention, and their association with restorative effects can be induced by encouraging meditation or the appreciation of greenery. Environmental characteristics of space, such as “spacious” and “places where people can gather”, contribute significantly to creating a spatial atmosphere where visitors can feel a sense of diversity and variation in the environment, and these will induce restorative effects by encouraging meditation, gathering, playing with children or enjoying outdoors. The environmental characteristics of social such as “paths with hard surfaces”, “entertainment” and “good general lighting” contribute more to the creation of a place where people can meet and interact with others, and these can induce restorative effects through the facilitation of gatherings, reunions, playing with children. Environmental characteristics of refuge such as “many bushes” and “watching people actively”, which contribute to the creation of environments offering a sense of shelter and protection, these can be used to induce restorative effects by promoting relaxation, dating and gardening. Environmental factors of serene such as “feeling safe” and “not crowded” contribute more to a peaceful atmosphere without disturbance and can result in a positive restorative effect by promoting the use of relaxation, inspiration and the appreciation of greenery.

These findings suggest changes in environmental characteristics can trigger the willingness of people to carry out certain activities and thus obtain a restorative effect. Hence, it broadens the boundaries of restorative environmental design considerations. For example, even though the social dimension generated the largest contribution to the restorative effect, the space dimension had the most comprehensive correlation pathways in the process of achieving restorative effect. This means that while the user reaps the greatest amount of benefit from the social dimension, the space dimension offers a greater variety of ways to benefit.

### Implications for Landscape Designers and Planners

The current lack of uniform standards guiding the planning and construction industry restricted future survival and development of PRGs in urban environments. The PSDs can comprehensively summarize each environmental characteristic in detail, and thus, they can be used both as a tool for analysis and as design guidelines for designers and planners.

This study has generated practical implications on how we can design public rooftop gardens targeted specifically around potential usage patterns and health benefits. For example, the PRGs should be designed with space or facilities for entertainment and exhibitions, paths with hard surfaces and general good lighting also need to be provided. These characteristics can induce restorative effects through the facilitation of gatherings, reunions, playing with children.

This study also suggested that providing a well maintained and not crowded space is necessary for human-place interaction. Serene atmosphere that makes people feel silent and calm, or feel safe is also important. These characteristics would be associated with restoration through promoting the use of relaxation, inspiration and the appreciation of greenery.

Nevertheless, the findings of this paper should be considered as a valid complement to the previous roofing studies that focused only on the aesthetic characteristics of plants, or as a design guideline for the PRG construction process, rather than a limitation in an absolute sense.

### Strengths and Limitations

This study has generated knowledge on the actual status of PRGs through collecting and describing the individual perception or behavior, and it is also the first attempt to concretize the characteristics of PRGs by combining the PSD as well the PRS in the context of China. In addition, this paper verified the pathway between the two mentioned above.

However, there are some limitations in this paper that need to be tackled in future research. First, the study samples applied in this paper are all over 2,000 m^2^. The ignorance of smaller-scale potential samples may affect the overall representativeness of PRGs. Then, the respondents were limited to adults aged 18 and above, ignoring the users of younger age, especially teenagers.

## Conclusion

This study revealed important findings about how usage patterns mediate the association between the PSD and PRS of users. It concluded that the five perceived sensory dimensions of social, refuge, serene, space and nature were induced into restorative effects through four patterns of use: retreat, nature touch, interpersonal interaction and family-bonding activities. It also found that family-bonding activities were the most influential mediator in achieving positive restorative effects in the PRG, with the remaining activities ranked as follows: retreat > nature touching > interpersonal interaction. A comparison of the path coefficients of the dimensions revealed that “fascination” brings about slightly more restoration than the rest, suggesting that more attention should be given to the setting of scenes related to it. The study also confirmed that five out of the eight environmental characteristics of PRGs had restorative effects, with the degree of influence being social > serene > refuge > nature > space. The findings increased knowledge on the pathways linked to PSDs and PRS amongst users of PRGs. However, in view of the diverse functions of these sites (PRGs), future researchers should investigate whether the PSDs/PRS pathways established will significantly differ under different PRGs. Given this study also shows that access to restorative benefits depends not solely on the characteristics of the environment but also on the specific ways in which they use it, this will also lead to the question if typologies of PRGs will induce specific usage patterns, that in turn strengthening furthers the pathway. In addition to this, individual needs or external conditions will also have a distinctive effect on usage patterns, such as age, gender, income level (7, 90–93)or thermal condition (94–99). Hence, additional moderating variables could be potentially introduced into this model to further improve the theoretical framework and conceptual model of the correlation pathway in PRGs in future research.

## Data Availability Statement

The original contributions presented in the study are included in the article/supplementary material, further inquiries can be directed to the corresponding author/s.

## Ethics Statement

Ethical review and approval was not required for the study on human participants in accordance with the local legislation and institutional requirements. Written informed consent for participation was not required for this study in accordance with the national legislation and the institutional requirements.

## Author Contributions

ZC and TZ: conceptualization. ZC and QD: methodology. ZC: investigation, visualization, and writing—original draft preparation. KKG and TZ: review. TZ and MZ: supervision. All authors have read and agreed to the published version of the manuscript.

## Conflict of Interest

The authors declare that the research was conducted in the absence of any commercial or financial relationships that could be construed as a potential conflict of interest.

## Publisher's Note

All claims expressed in this article are solely those of the authors and do not necessarily represent those of their affiliated organizations, or those of the publisher, the editors and the reviewers. Any product that may be evaluated in this article, or claim that may be made by its manufacturer, is not guaranteed or endorsed by the publisher.
