# Supplementary material for: Using Structural Equation Modeling to Examine Pathways Between Environmental Characteristics and Perceived Restorativeness on Public Rooftop Gardens in China
Source: Front Public Health. 2022 Feb 24;10:801453. doi: 10.3389/fpubh.2022.801453 (PMC8907599; doi:10.3389/fpubh.2022.801453)
Supplement: Supplementary file 1 [file Table_1.DOCX]

Table S1 Ratings of the PSDs and Intraclass Correlation Coefficient(ICC) in each PRG of users

|  | Nature  Ratings/ ICC | | | Space  Ratings/ ICC | | Culture  Ratings/ ICC | | Social  Ratings/ ICC | | Refuge  Ratings/ ICC | | Serene  Ratings/ ICC | | Prospect  Ratings/ ICC | | Rich in species  Ratings/ ICC | | |
| --- | --- | --- | --- | --- | --- | --- | --- | --- | --- | --- | --- | --- | --- | --- | --- | --- | --- | --- |
| Site 1 | 2.81  Low | 0.832^c^ | | 4.07  High | 0.915^c^ | 3.68  High | 0.873^c^ | 3.25  Medium | 0.849^c^ | 3.26  Medium | 0.831^c^ | 3.44  Medium | 0.777^c^ | 3.33  Medium | 0.791^c^ | 2.00  Low | 0.790^c^ | |
| Site 2 | 2.99  Low | 0.786^c^ | | 3.80  High | 0.880^c^ | 4.19  High | 0.856^c^ | 3.79  High | 0.877^c^ | 3.35  Medium | 0.854^c^ | 3.51  Medium | 0.760^c^ | 3.44  Medium | 0.857^c^ | 2.90  Low | 0.703^c^ | |
| Site 3 | 3.11  Low | 0.898^c^ | | 3.75  High | 0.869^c^ | 3.04  Low | 0.889^c^ | 3.63  High | 0.877^c^ | 4.05  High | 0.760^c^ | 4.01  High | 0.726^c^ | 2.97  Low | 0.711^c^ | 2.90  Low | 0.667^c^ | |
| Site 4 | 3.50  Medium | 0.854^c^ | | 3.14  Medium | 0.844^c^ | 2.05  Low | 0.793^c^ | 2.54  Low | 0.870^c^ | 3.50  Medium | 0.857^c^ | 3.96  High | 0.657^c^ | 3.19  Medium | 0.852^c^ | 2.60  Low | | 0.472^c^ |
| Site 5 | 2.83  Low | 0.880^c^ | | 3.40  Medium | 0.850^c^ | 3.13  Medium | 0.848^c^ | 3.93  High | 0.891^c^ | 3.69  High | 0.825^c^ | 3.19  Medium | 0.739^c^ | 2.87  Low | 0.735^c^ | 3.80  High | | 0.810^c^ |
| Site 6 | 3.60  Medium | 0.900^c^ | | 4.25  High | 0.854^c^ | 2.72  Low | 0.870^c^ | 3.82  High | 0.880^c^ | 4.00  High | 0.778^c^ | 3.85  High | 0.840^c^ | 3.10  Low | 0.894^c^ | 3.80  High | | 0.710^c^ |
| Site 7 | 3.43  Medium | 0.870^c^ | | 3.64  High | 0.863^c^ | 3.49  Medium | 0.871^c^ | 3.73  High | 0.885^c^ | 3.25  Medium | 0.805^c^ | 3.27  Medium | 0.821^c^ | 4.02  High | 0.833^c^ | 3.70  High | | 0.755^c^ |
| Site 8 | 4.48  High | 0.839^c^ | | 4.78  High | 0.853^c^ | 2.97  Low | 0.861^c^ | 3.67  High | 0.864^c^ | 4.14  High | 0.733^c^ | 3.87  High | 0.826^c^ | 3.28  Medium | 0.825^c^ | 4.60  High | | 0.782^c^ |
| Site 9 | 3.37  Medium | 0.857^c^ | | 3.42  Medium | 0.840^c^ | 3.23  Medium | 0.788^c^ | 3.10  Low | 0.889^c^ | 3.77  High | 0.860^c^ | 3.61  Medium | 0.864^c^ | 4.31  High | 0.863^c^ | 3.40  Medium | | 0.661^c^ |
| Site10 | 2.53  Low | | 0.885^c^ | 3.17  Medium | 0.796^c^ | 2.93  Low | 0.869^c^ | 2.63  Low | 0.674^c^ | 3.16 Medium | 0.761^c^ | 2.90 Low | 0.750^c^ | 2.52 Low | 0.850^c^ | 2.30  Low | | 0.813^c^ |
| Site11 | 3.33  Medium | | 0.867^c^ | 3.10  Low | 0.820^c^ | 3.13  Medium | 0.719^c^ | 3.20  Medium | 0.819^c^ | 3.29 Medium | 0.744^c^ | 3.74 High | 0.816^c^ | 3.49 Medium | 0.821^c^ | 2.70  Low | | 0.749^c^ |
| Site12 | 2.46  Low | | 0.643^c^ | 2.76  Low | 0.770^c^ | 2.48  Low | 0.874^c^ | 2.79  Low | 0.779^c^ | 2.89 Low | 0.817^c^ | 3.66 High | 0.780^c^ | 2.52 Low | 0.844^c^ | 2.30  Low | | 0.746^c^ |

**Psd ratings: 3.63-4.78= High 3.13-3.61= Medium 2.00-3.11 =Low**

**Average Measures used in this paper to represent ICC，0 means untrusted , 1 means completely trusted. It is generally believed that the ICC below 0.4 indicates poor reliability, while that above 0.75 indicates good reliability.**
